# The Devil Is in the Detail: Challenging the UK Government’s 2019 Impact Assessment of the Extent of Online Marketing of Unhealthy Foods to Children

**DOI:** 10.3390/ijerph17197231

**Published:** 2020-10-02

**Authors:** Mimi Tatlow-Golden, Daniel Parker

**Affiliations:** 1 Faculty of Wellbeing, Education and Language Studies, The Open University, Milton Keynes MK7 6AA, UK; 2 Living Loud, Brighton, Sussex BN1 3SU, UK; danparker@livingloud.org.uk

**Keywords:** advertising, marketing, regulation, policy, children, adolescent, digital, online, TV

## Abstract

Background: How much unhealthy marketing do children see on digital devices? Marketing of unhealthy food and beverages has long been identified as a factor in children’s preferences, purchase requests and consumption. Rising global obesity mandates States to craft environments that protect children and young people’s health, as recommended by the World Health Organization, among others. However, assessing the impact of marketing restrictions is particularly challenging: the complexity of digital advertising markets means that measurement challenges are profound. In 2019, the UK Department of Health published an Impact Assessment that applied a novel method aiming to calculate costs and benefits of restricting unhealthy food and beverage advertising on digital devices (planned for implementation by 2022). It estimated UK digital unhealthy marketing to children at 0.73 billion advertising impressions annually, compared to television impacts of 3.6 billion. Aim and Method: We assessed this conclusion by reviewing the UK Department of Health/Kantar Consulting’s Online Baseline Methodology (the “Government Model”). We examined the model’s underlying premise and specified the seven analytic steps undertaken. For each step, we reviewed industry and academic evidence to test its assumptions and the validity of data applied. Results: We found that, in each step, the Government Model’s assumptions, and the data sources selected, result in underestimates of the scale of digital advertising of unhealthy foods—at least tenfold, if not substantially more. The model’s underlying premise is also problematic, as digital advertising *spend data* relate poorly to digital advertising *exposure*, leading to further underestimation of market scale. Conclusion: We conclude that the Government Model very substantially underestimates the impact of digital unhealthy food advertising restrictions on health. This analysis has relevance for global policy and for the impact of regulation on children’s health and well-being.

## 1. Background

Childhood obesity has become a global issue in the 21st century, with children worldwide affected by the “double burden” of malnutrition and associated consequences for children’s health and well-being [1,2,3]. Marketing for unhealthy, ultra-processed foods and beverages (usually conceptualised as those high in saturated fat, sugar or salt; henceforth, “unhealthy food”) is well evidenced as a factor in children’s food preferences, purchases and eating [3,4,5,6,7]. Marketing exposure prompts the eating of extra items, raising calorie intake that is not compensated for later, and this has a sustained impact over time [5,6]. Thus, there are long-standing calls to protect children comprehensively from food marketing by the World Health Organization, UN Special Rapporteurs, and many civil society bodies [7,8,9]. Lately, calls to regulate food marketing on digital devices (henceforth: digital marketing) have been growing apace, and these have also specified the need to protect not just younger children but teens as well [9,10,11,12]. This is partially due to their extensive use of digital media but also because it is increasingly understood that advertising and media literacy do not provide the protection that was earlier assumed, and that this is even more the case where the manipulative strategies of digital marketing are considered [2,12].

When considering regulation, a key requirement for policymakers is to be in a position to assess the impact of any proposals. To do so requires identifying children’s *exposure* to advertising. Unlike in television, in digital media this presents a “wicked problem”, created by the business model of the internet. This has evolved this century to be almost exclusively advertising-funded, supported by an extraordinarily complex advertising technology ecosystem that extracts user data and auctions ad space (in millisecond online auctions) to users whose profiles indicate they have an interest in the product [2,13,14]. Where formerly advertising was contextual—delivered to settings where audiences were assumed to be interested—now it is carried out by “programmatic” automated processes [13,14]. Every internet user, including children, sees a different set of ads, depending on their preferences and previous engagement as recorded by the device, sites and apps they use. Furthermore, social media platforms do not permit review of their advertising and targeting practices, meaning that digital advertising activity takes place within a “black box”, inaccessible to researchers and even regulators [15].

In 2018, the UK government’s Childhood Obesity Plan Chapter 2 was published [16], following from a sugar tax introduced three years earlier. Among its proposals was a 9 p.m. “watershed” for marketing unhealthy food and nonalcoholic beverages, in which such advertising would be prohibited except between 9 p.m. and 5:30 a.m. on TV, in digital media, or both. In 2019, a proposal for these further advertising restrictions was announced [17], accompanied by a 2019 Department of Health and Social Care (DHSC) and Department of Culture, Media and Sport (DCMS) Consultation [18] on restricting unhealthy food and drink advertising on TV and online, that sought evidence from academics, civil society, industry and other interested parties regarding the potential social, business and health impact of such advertising restrictions.

To inform the 2019 consultation, the two government departments published an Impact Assessment (IA) [19], for which Kantar Consulting was commissioned to assess potential costs and benefits of four policy options—health benefits for children and the economic impact on advertisers, broadcasters and the food and drink industry (IA No: 13013). The DHSC [18,19] applied consumption-related effects seen in experimental consumption studies to estimated advertising exposure and concluded that applying a ban on advertising unhealthy foods before 9 p.m. on TV and online “could remove around 8 billion calories per year in total from children’s (across all 4–15-year-olds) diets by direct influence on children’s consumption” (p. 6) [18].

In its report, the DHSC pointed out the challenges of making such assessments. Even for television, where advertising is transparent, the IA faced the challenge of factoring in children’s changing viewing habits and a wide range of channels, as well as distinguishing products high in saturated fat, sugar or salt (HFSS) from non-HFSS product advertising and brand from product advertising. It concluded that there were approximately 3.6 billion HFSS child impacts in 2017 on TV, 2.6 billion before the watershed (p. 26) [18].

With regard to digital media, the measurement challenges are far greater. The advertising market is extremely complex, opaque and disaggregated [13,14,18,19]. As the IA states [19], there is “lower transparency in the system - reflecting the lack of comprehensive independent public data, widespread personalisation of advertising, and the sheer scale of the online advertising landscape” (p. 26). Kantar Consulting was thus commissioned to develop an “unprecedented” Online Baseline Methodology (p. 27; henceforth, the Government Model. See Annex D, pp. 124–127) [19].

The Government Model sought to answer this question: How much online advertising for unhealthy food and drink are children in the UK potentially exposed to annually? As the Government Model notes (p. 126), there is no online equivalent of the UK Broadcasters’ Audience Research Board (BARB) that can report digital advertising impressions for particular audiences. Given the known challenges of measuring children’s actual digital advertising exposure, the Government Model sought to assess this based on the premise that advertising *spend* data can be used to estimate digital advertising *exposure*. Applying a sequence of assumptions and estimates to spend data, the Government Model concluded that children in the UK are exposed to 0.73 billion HFSS impressions annually in digital media, or one-fifth of the HFSS advertising they see on television.

In this paper, we seek to assess the accuracy of this conclusion. To do so we interrogate the method applied in the Government Model. Step by step, we specify its assumptions, calculations, and the data sources relied on, reviewing grey/industry and academic literature to examine whether its conclusion of children’s digital advertising exposure is safe. In doing so we aim to contribute to the global discussion about monitoring children and young people’s exposure to marketing of unhealthy foods, and to consider the potential impact of legally binding measures to restrict it. The aim of the paper is thus to assess the validity of the UK 2019 Impact Assessment’s estimates for children’s exposure to digital marketing.

## 2. Methods

The Impact Assessment’s Online Baseline Methodology (see Annex D, pp. 124–127) [19], developed by Kantar Consulting (Government Model), was analysed to specify each of its assumptions, inferences and data sources step by step. For clarity, we first formulated these as a sequence of seven implicit questions that guided the methodology. Next, to test the validity of each answer in the model, we consulted a range of marketing, media and food/drink industry sources. These included standard digital marketing data sources such as the Internet Advertising Bureau (IAB), WARC (an industry research body), and eMarketer, as well as academic and grey literature about advertising effects, and industry literature about the impact of digital marketing. We sought alternative data sources and reviewed the literature for accounts of the validity, scale, scope and nature of the digital advertising economy, food marketing and digital marketing to young people. The review was therefore not a general one but specifically focused on answering the seven implicit questions in the method applied. With this, we examined the Government Model’s premises, calculations and data used, to draw conclusions about the accuracy of the IA’s estimates of children’s digital exposure.

## 3. Results and Discussions

In this section, we first summarize the overall pathway taken in the Government Model. Next, we address its base assumption, discussing why it is problematic and how it must lead to major underestimates of exposure. After that, we consider each of the assumptions and inferences of the Government Model’s analysis, step by step.

### 3.1. Government Model’s Premises, Assumptions and Calculations

To estimate the annual unhealthy digital ads to which children are exposed, in the absence of data about actual advertising exposure, the Government Model applied a sequence of assumptions and inferences. The base assumption was that marketing *spend* can be applied to estimate digital ad *impressions*. The model therefore started with spend data for all-channel food and drink advertising in the UK. Next, it sought industry data to estimate the proportion of this spend in digital media. The food and drink annual online spend estimate was then broken down by estimates of the proportions of different digital ad formats (there are at least 11 different types of online advertising, described below). The overall UK online food and drink spend estimate was then divided up, using these ad format estimates and their costs. This yielded an estimate of the total number of UK food and drink ad impressions. Next, industry data for one type of digital ad were applied to infer the likely overall proportion of online ads for HFSS products. Finally, from this online HFSS proportion, a further data source was applied to infer the proportion of HFSS impressions likely seen by children.

In Table 1, in the left-hand column, we state each step undertaken in the Government Model as a question, summarizing its assumption, data source or calculation made, and state the model’s conclusion for that step. The right-hand column summarizes the findings of our review. In the following sections, we address each of these steps and consider its validity.

### 3.2. Inaccuracies in the Government Model

In the following sections, we specify each error we identified in the Government Model, laying out analysis and the supporting evidence.

#### 3.2.1. Base Assumption Error: Digital Ad Spend Is Mistakenly Equated with Reach

The base assumption of the Government Model is that the scale of children’s exposure can be assessed by applying digital marketing spend metrics to estimate the size of the UK’s digital advertising market and the number of advertising impressions. Although it may seem logical to draw on advertising spend data to estimate total advertising exposure, this does not apply well to digital advertising. In this section, we explain the two main reasons for this: first, much digital spend is not captured by existing industry metrics; second, spend does not translate simply into reach achieved.

##### Industry Data Do Not Capture All Spend

Industry publications state that industry spend data only capture a small proportion of digital ad spend. As advertising and marketing experts Les Binet and Peter Field noted in 2017, “media auditors such as Nielsen do try to measure online adspend, but it is extraordinarily difficult, and it is widely acknowledged that they only capture a fraction of what clients are spending online” [20], p. 38. Because of this uncaptured spend, any subsequent analysis is working from an unspecified subset of children’s digital marketing exposure.

What does the uncaptured spend go towards? A key element is “brand activations”—estimated to be three times the size of the conventional digital advertising market [21]. Brand activations are not typical advertising, as they do not interrupt other content, but rather integrate marketing messages into the digital experience, content and conversation (see Box 1). Case studies and brand testimonials indicate that these novel techniques are widely employed by HFSS products and brands.

Box 1 **Examples of novel brand communications not captured by standard digital spend metrics.**

**Influencer marketing**, a growing strategy, employs influential people in social media channels to promote brands, products or services. Food is its second-most-active industry [22]. Influencers are particularly effective with children and young people: ads with a celebrity presence resulted in a 16% greater impact on brand awareness compared to those without, and “Gen Z” are significantly more receptive than others to content featuring celebrities and social media celebrities [23]. The scale of the global influencer industry is debated [24]; estimates for 2020 suggest 6.12 billion brand sponsored influencer posts annually and industry market estimates range from $2 to $10 bn billion [25,25,26,27].

**Advergaming** uses a brand or product within the design of game play, such as Pokemon Go, which has driven 500 m visitors to sponsors’ locations such as McDonald’s [28] or the McDonald’s integration into the Farmville game used by 215 m people on Facebook [29].

**Social media**: Food and drink companies invest in building channels on sites such as Facebook and Instagram that allow them to achieve major reach with no or minimal promotional spend. As an example, Coca-Cola has over 100 m followers on Facebook, meaning that their posts on their channel, whether promoted (and paid for) or not, have the potential to achieve very extensive reach. In social media, there are multiple ways that brands can achieve exposure and advertising can be identified as paid, owned or earned: “earned” ads come from peer recommended likes, shares and other interactions with advertising content; “owned” ads involve the brand publishing its own adverts (i.e., posts); and “paid” ads are where the brand pays, e.g., to amplify a campaign.

Coca-Cola’s Chief Digital Officer states that they aim to take advantage of how consumers “participate, actively and co-create” as “experience makers”, something that happens “tens of thousands of times a day because of their love and their community with the brand” [30]. McDonald’s defines social media as a “two-way street allowing dialogue, kinship and collaboration” [31], thereby recruiting teens to engage, to feel like part of a junk food brand family, and to become part of a network promoting their products. These activities show brands aiming to become integrated into young people’s social interactions and extending far beyond the notion of advertising as information.

**Event sponsorship**: A further brand activation approach that creates visibility without direct advertising spend is sponsorship of sports and entertainment events. The energy drink brand Red Bull is a key user [32], developing sports, entertainment and lifestyle content, hosting live events, purchasing sports teams, sponsoring emerging music artists and more, with almost no product content—their CEO has stated “we believe that we can activate through events and great content, and that’s where we’d rather spend our money … we do a lot of things where you’re like, ‘wow, that has nothing to do with the brand’, but it still really makes good content” [32]. This achieves millions of social media followers and heavy engagement. Brands that advertise heavily also use this approach; for example, Coca-Cola’s Olympics sponsorship earns widespread sharing in social media, such as the #thatsgold hashtag for the 2016 Games that is still in use 4 years later [33].

There is therefore very extensive food-and-drink-related digital marketing spend and activity that is not captured by existing spend metrics. This is significant, given children and young people’s extensive digital media use: in the UK, 69% of 12–15-year-olds, and 18% of 8–11-year-olds have a social media profile, and YouTube is the most viewed site by children in general [34]. We consider evidence for the impact of digital marketing in the General Discussion below.

##### Spend Does Not Reflect Reach In Digital Marketing

The second reason why spend data are a poor proxy for exposure is that, even where spend is captured, it does not directly correspond to “reach” (i.e., how many people see a given piece of digital marketing content). In television and other traditional media, spend correlates directly with reach, as ads for larger audiences cost more. In digital media, however, spend and reach are not clearly associated. Spend can achieve specified reach, but brand-owned content, network effects and virality can transform this. Brands can create their own content on their social media accounts. They encourage social media users to “like” content and share with friends, boosting its visibility via social media algorithms. In these ways, small or even no spend can achieve widespread reach and impact. Indeed, the entire digital ecosystem is premised on engaging media users, including children, to disseminate content [2,35,36]. Social media earnings reports also indicate the disconnect between spend and reach. For example, in Q4 2018, Facebook reported that ad prices decreased by 2% but impressions were up by 34% [37].

Insights into the spend/reach disconnect can be glimpsed when marketers submit information to industry awards [38,39]. The WARC awards 2018 (p. 5) noted that lack of budget was no hindrance to success: campaigns with “no or negligible budget” that successfully tap into “news, memes and broader cultural trends” can be widely shared [39]. Successful campaigns can magnify reach, doubling or quadrupling the expected campaign audience, or more, particularly where they employ originality and humour [38]. Examples are given in Box 2.

Box 2 **Campaigns for unhealthy foods gaining extensive reach showcased at WARC annual industry awards.**

A UK KFC (Kentucky Fried Chicken) “Dirty Louisiana” Burger campaign used humour to subvert the rising trend for “clean eating” and promote its “indulgent beast” of a burger with chicken, double cheese, a hash brown and three sauces. It launched a fake blogger (“Figgy Poppleton-Rice”), fake fans, and fake recipes (e.g., a raw cauliflower and ice cube burger). A full campaign spend of £200,000 was expected to generate 17 m impressions and 3 m views. The campaign instead achieved 74 m impressions and nearly 20 m views of the campaign video, reaching over 1 in 3 internet users in the UK alone [40]. The humorous campaign benefited from earned media to generate four times as many impressions and ten times as many views as anticipated by the original spend.

In 2017, Fanta engaged with teens on Halloween. A London artist’s spooky character designs and increasingly creepy Instagram, Facebook and Snapchat content leading up to October 31 spread the message that Fanta fuels fun. Product “Snapcodes” provided access to Halloween-themed lenses or filters. Halloween-themed social content for all markets and Halloween-themed content piloted a Fanta “Buy Now” button on Facebook in the UK. This proved to be one of Snapchat’s most successful brand activations, achieving significant rises in sales in several Western European countries with 25 million unique users interacting with the campaign, 137 m impressions; 1.14 m on-pack Snapcodes were unlocked and users then engaged with this content for an average of 37 s, one of the highest in Snapchat’s history [41].

These growing forms of communication created and managed by HFSS brands are very appealing to children and young people and are excluded from consideration in the Government Model. This further demonstrates the fallacy of relying on advertising spend to assess the extent of HFSS digital advertising to which children are exposed.

Having identified the weak underlying premise of the Government Model, which must result in an underestimate of the digital market, we next turn to its steps and assumptions, examining each in the context of further industry data.

#### 3.2.2. The Scale of Digital Marketing Is Underestimated

***Question 1:*** *Of overall (all-channel) food and drink advertising, what proportion is represented by digital?*

The Government Model (p. 124) uses Nielsen Ad Dynamix data as the source to estimate that digital food advertising is 8% of all-channel food advertising; digital drink advertising is 5% of all-channel drink advertising (Nielsen/WARC) [19]. These are really very low percentages and are not credible in light of the scale of the UK digital advertising market. For example, the President of Kellogg’s Snack has stated that Kellogg’s spends 60–70% of their overall marketing budget on digital platforms [42].

Examining Nielsen Ad Dynamix data for all sectors, it can be seen that this data source estimates all-sector UK digital advertising spend to be just 18% of all-channel advertising spend. Again, this is very low compared to other digital industry data. Indeed, Nielsen’s own data and statements undermine this data source. Nielsen’s 2018 CMO report [43] states that digital ad spend has eclipsed traditional channels; that only one in five respondents reported spending less than 20% of advertising budget on digital.

Furthermore, multiple recognized industry sources undermine the credibility of the Nielsen Ad Dynamix figure. These estimate the UK all-sector digital ad spend proportion as ranging from 52–66%, 3 times greater than the Nielsen all-sector estimate of 18%:

- Advertising Association /WARC: Figures released by these advertising bodies place digital spend across all sectors at 52% of total advertising expenditure [44];
- DCMS Online Advertising in the UK Report 2019: This government-commissioned report cited Internet Advertising Bureau (IAB) data: in 2017, internet advertising overtook all other forms of advertising (television, press, radio, cinema and outdoor) combined, a 52% share of total advertising spend [45];
- Group M: Digital spend is 60% [46];
- eMarketer UK Digital Ad Spend Report: In 2019, digital had already accounted for the majority of media ad spending in the UK for several years—in 2019, its share would be 66.4% [47].

This discrepancy between Nielsen’s Ad Dynamix digital data and all these other industry data sources (even including other Nielsen data) may be explained by the method with which Ad Dynamix estimates advertising expenditure: spot monitoring [48]. Spot monitoring involves viewing and noting the ads on display in any given location at a given moment. It is relevant for placement methods in news or television media. However, digital advertising is now delivered via profile targeting in a programmatic system —each user sees different ads on the same page based on their digital profile, device and app use history, and preferences. Programmatic advertising now accounts for 87% of UK ad spend in the UK [49] and 65% of digital spend globally [50], and a monitoring method that cannot account for it means that spend cannot be captured accurately.

Nielsen Ad Dynamix is therefore an outlier in its digital estimates. It places overall UK digital marketing at 18% of all-channel spend, whereas multiple aligned credible industry data sources estimate all-sector UK digital spend at 52–66%. In this context, relying on Nielsen Ad Dynamix percentages of digital food and drink spend affects the credibility of this estimate and hence of all the figures that follow. Multiplying by a factor of 3 would be a reasonable corrective.

#### 3.2.3. Food and Drink Ad Spend Is Underestimated

***Question 2:*** *Of UK all-channel advertising spend, how much is spent on food and drink?*

The Government Model (p. 124) states that of UK all-channel advertising spend in 2016, food was £927 m (5% of all-channel spend) and drink £314 m (1.6% of all-channel spend), a total of £1,241 m (Group M and Statista) [19]. These figures do not capture grocery retail and restaurant marketing and are thus likely to underestimate total spend on marketing unhealthy products. We explain this further in the analysis for Question 3 below.

#### 3.2.4. Digital Food and Drink Ad Spend Is Therefore Underestimated

***Question 3:*** *What is the digital food and drink advertising spend total for the UK?*

The Government Model next applied the Nielsen Ad Dynamix proportions from Question 1 (8% and 5% digital advertising for food and drink), to the data for Question 2, i.e., food (8% of £927 m) as £74 m and drink (5% of £314 m) as £15.7 m. The model therefore estimated UK digital food and drink spend as £89.7 m (p. 124) [19].

If instead the widely accepted figure of 52–66% of total advertising spend on digital (identified above for Question 2) were applied to the combined total overall spend for food and drink alone (£1,241 m—a conservative estimate as it disregards grocery and restaurants), this would result in an estimated total annual digital food and drink spend of £645 m–£819 m per year, up to 9 times greater than the Government Model estimate.

How does this range compare with other industry data? Can a difference of such a magnitude be supported? We explored alternative data sources to test our assumption further.

We consulted the IAB (Internet Advertising Bureau) annual UK Adspend Study 2018 [51]. The IAB Adspend study has been the official digital advertising data source for the Advertising Association since 1997. Adspend is conducted quarterly for the IAB in multiple regions by PwC. Rather than relying on spot monitoring, it uses actual bookings data submitted directly to PwC from media owners, intermediaries and agencies—based on data from “desktop and mobile online advertising revenues from websites, commercial online services, ad networks and exchanges, mobile devices, and email providers, as well as other companies selling online advertising”’ Although PwC does not audit the data, its sources mean that it is considered a “reasonable measurement”, (p.1) [52] and industry views it as a more credible source of spend data than Nielsen Ad Dynamix. This study [51] tells a very different story.

The IAB Adspend Study provides data for three categories that include food and drink: Consumer Goods (CG), retail and restaurants. To extract food and drink data (i.e., food and drink; grocery retail; and quick-service restaurants) from these broader categories, we consulted other industry data to estimate their proportions within each sector. We detail these steps in Box 3. In sum, the IAB data generate a total estimate for UK digital advertising spend for food and drink (including grocery retail and quick-service restaurants) of £731,000,000. Note that this sits within the range estimated above (£645 m–£819 m) and thus is supported by other industry calculations. This is over 8 times greater than the Government Model estimate.

Box 3 **An alternative calculation, from industry data, of UK food and drink digital advertising spend.**

The IAB Adspend report [51] includes advertising spend data for three sectors within which food and drink can be found: CG (Consumer Goods), retail and restaurants. To estimate food and drink (within CG), grocery (within retail) and quick-service restaurants (within restaurants), we identified their proportions from the following industry data:

**FMCG digital advertising spend**: The IAB reports CG spend as 11.54% of the £4,307 bn entire UK digital display market (i.e., online advertising excluding search and classifieds), thus it can be estimated as £605 m. Statista reports food and drink spend [53] to be 82% of FMCG (Fast-Moving Consumer Goods, another term for CG). Thus, food and drink can be estimated as £496 m.

**Retail digital advertising spend**: The IAB reports spend for retail as 10.9% of digital display, thus it can be estimated as £572 m. This includes grocery and non-grocery. Ebiquity’s The Advertising Report [54] allows for an assessment of the proportion of grocery advertising spend, as it lists all-channel UK retail advertising spend as £1.81 bn. Of this, Tesco spends £73.9 m, Asda £59.5 m, Morrisons £51.8 m, Sainsburys £44 m, Aldi £48.8 m and Lidl £70.5 m. The total for these six major grocers is £348.9 m, which constitutes 19.3% of all-channel retail ad spend. Applying this percentage of 19.3% to the IAB digital spend data for retail, this estimate results in a retail/grocery spend of over £110 m/year online.

**Restaurant spend:** The IAB data list 2.38% of digital display advertising as restaurants, thus it can be estimated as £125 m. As an example of the scale of quick-serve restaurant advertising, McDonald’s alone spent £122 m on all channel advertising in the UK in 2018 [55], rising to £151 m in 2019 [56]. We therefore estimate that this spend can be allocated to quick-serve restaurants and those featuring less-healthy offerings almost in its entirety, supported by the IAB definition (see p. 64; examples it gives are Dunkin Donuts, Pizza Hut and McDonald’s). Therefore, we estimate restaurant spend as £124 m.

**Total: £730 m**

In sum, for Questions 2 and 3 alone, major underestimates have been identified that suggest that the spend estimate is undervalued by a factor of 8. The subsequent steps of the Government Model introduce still further potential underestimates of advertising spend and impressions.

#### 3.2.5. The Digital Advertising Format Most Frequently Used by Food and Drink Is Miscalculated

***Question 4:** What online advertising formats does online food and drink consist of?*

Online advertising consists of many different formats, e.g., native advertising, display banners desktop, display banners mobile, search, etc. (see Box 4 for definitions). The Government Model presents data (cited as being from Group M investment) on how UK online advertising is distributed (the “splits”) across different online formats (pp. 125–126) [19]. This finds that across all sectors, 57.7% is allocated to search advertising. The Government Model applies the assumption that splits for food and drink mirror all-sector splits. Yet the IAB 2018 Adspend report [Section 2.4, pp. 50–55) demonstrates that there are substantial differences in the patterns of industry sector spending by digital advertising format. For example, consumer goods (which includes food and drink) account for the smallest proportion of spend on paid search (1.55%) but the largest proportion on total display (11.54%), social networking sites (13.52%) and non-social (10.86%). As food and drink dominates the overall consumer goods category, we believe it is reasonable to infer that its digital spend splits follow a similar pattern—and does not reflect the splits for all sectors applied by the Government Model.

Box 4 **Definitions of major types of online advertising formats and their all-sector splits in the UK.**

**Search** (57.7%): Paid-for listings in search results, e.g., sponsored links on Google.

**Native** (10.2%): A form of display advertising that is integrated into the surrounding content, such as in a social media feed.

**Display banners desktop** (8.9%): Ads for desktop devices shown in a distinct section of a webpage or app, e.g., at the top or on the side. Banners delivered to mobile devices are counted separately (see below).

**Display video outstream** (8.9%): Video ads shown in a non-video context.

**Display video pre-roll** (6.7%): Video ads shown before video content.

**Display banners mobile** (4.1%): See Display banners desktop above.

**Sponsored** (1.2%); **Other display** (1%)

Source for all-sector split data: DHSC/DCMS 2019 [19], pp. 125–126. Citing Group M Investing. Source for definitions—adapted: Plum Consulting report for DCMS (p.6) [45].

#### 3.2.6. The Total Number of Food and Drink Online Ads Is Underrepresented

***Question 5:*** *How many UK food and drink online ad “impressions” are there in total?*

The Government Model next takes the splits applied in Question 4 and applies Cost per Mille (CPM) data (a charge for each thousand times an advert is displayed) for different advertising formats to estimate the number of ad impressions overall. The outcome is an estimate that the UK online food and drink advertising market consists of 22 bn impressions annually (p. 126) [19].

In addition to the inaccurate assumption we identified for Question 4, there are three further issues with the approach taken to answer Question 5. These are detailed in Box 5.

In sum, first, the cost measure applied is out of date as CPM is fading in use; Cost Per Click (CPC, a charge for when a user clicks on an ad) is more widespread. Second, having accorded over half of the splits in the previous step to search, the Government Model now discounts it completely. Although we noted above that search represents a small proportion of food and drink online advertising spend, the decision to disregard it entirely is likely to have some impact, albeit relatively small compared to other factors. Much more significant however—and as noted regarding Question 4—is that, third, food and drink online advertising favours display advertising, particularly native, in social media. This is significant because native is 16 times cheaper than the desktop display CPM cost applied in the Government Model (see Box 5 for details). The allocated spend would therefore buy more impressions—once again indicating that the scale of impressions achieved online from spend on food and drink has been very substantially underestimated. Yet again, this suggests that each step of the chain of premises and inferences in the Government Model has the effect of significantly under-calculating exposure.

Box 5 **Estimating the number of digital ad impressions: Issues with the Government Model.**

**CPM or CPC?**

Less and less advertising today is sold using a CPM model, i.e., by measuring the number of times an ad is displayed. Instead, CPC (Cost per Click, a charge each time the advert is clicked) is now prevalent and heavily favoured by Facebook and Google [57,58]. The use of an outdated and little-used model for correlating spend and exposure of digital advertising undermines the credibility of the model. The implications of this failing are unknown.

**Discounting search**

Another issue follows on from the use of CPM rather than CPC. Search advertising is sold on a CPC basis, so by using a CPM model in the Government model, Kantar cannot attribute any exposure to search advertising. Kantar therefore makes the surprising decision to attribute zero exposure/impressions to search, discounting search to zero. Whether online food and drink search advertising is 57.7% (as inferred from the all-sector splits and applied in the Government Model) or much less (as inferred from the IAB data for CG advertising), it appears that the Kantar assumptions and modelling result in excluding a portion of advertising, albeit a small one.

**Overpricing food and drink advertising**

Perhaps most significantly for this question, the Government Model uses the desktop display banners cost of £8.05 per 1000 impressions to estimate the number of impressions overall. Yet in social media, native (advertising in the content feeds of social media) is much more prevalent than display banners—and is 16 times cheaper: 50p per 1000 impressions. Therefore, for native, the same spend gives 16 times more exposure. As noted above, IAB data show that CG favours social media channels for display rather than banners (68% of display spend, compared to 58% for all sectors). As the ratio of native-to-display banners used is wrong, once again, the assumptions made in answering Question 5 will underestimate the overall exposures.

#### 3.2.7. The Number of Unhealthy Ads Is Underrepresented

***Question 6:*** *How many UK online HFSS ads are there?*

Next, the Government Model estimated the proportion of the 22 billion food and drink advertising impressions that are HFSS ads. To do this, the model refers to ComScore data (p. 127) [19]. ComScore measures desktop banner display ads. They record 59% of food and drink ads as HFSS. The model notes that “the team have assumed that the same split of HFSS advertising observed in the desktop display sample applies to all other impact-bearing digital channels (desktop and mobile display, video and native advertising)” (p. 127). This percentage was therefore applied to the 22 billion estimated impressions, resulting in an estimate of 13 billion digital HFSS impressions (p. 126) [19].

Again, the evidence indicates that this represents an underestimate. Advertising online is device-specific. Different patterns of advertising go to mobile versus desktop/laptop devices. ComScore data apply only to desktop/laptop and only to one advertising format (banner display advertising), so they represent only 9% of overall online advertising spend. However, as noted above, desktop banner display is *not* a key marketing format for food and drink online.

Food/drink advertising favours social media channels which (i) employ native advertising and (ii) are predominantly accessed by mobile and tablets. Indeed, desktop/laptop data may particularly underrepresent HFSS advertising which is likely to be targeted to youth audiences via their preferred devices—mobile/tablet [59]. For example, 75% of 5–15-year-old children in the UK use tablets; desktop/laptop use is in decline. Half of children have a social media profile by age 12 (despite being underage), and 100% by age 15 [34]. Consequently, the ComScore data applied in this assumption have potential for a significant margin of error.

We do not have data that allow us to estimate by what kind of a factor this diverges from the Government Model estimates; however, it is likely to be a substantial underestimate.

#### 3.2.8. Child Exposure to Unhealthy Ads Is Underestimated

***Question 7:*** *What proportion of UK HFSS online ads are seen by children?*

The final step estimates the proportion of the 13 billion HFSS impressions seen by UK children. Here, the Government Model draws on Kantar’s in-house CrossMedia tool to estimate the proportion as 5.3%. This results in an estimate of 0.73 bn HFSS impressions seen by children in UK annually.

Again, our assessment is that the data used are likely to lead to an underestimate. Kantar’s site states that CrossMedia uses “integrated techniques to tag and measure web, video and apps” but that this tool is “still in its early days” [60]. In Annex D of the Impact Assessment document, Kantar indicates that CrossMedia employs panel survey data, drawing exposure inferences from “respondent-level answers to surveys asking about socio-demographic features and media behaviour patterns” (p. 127) [19]. Rather than a measure of advertising exposure, it is therefore a survey measure of media/website consumption. Yet 87% of online advertising in the UK is placed programmatically based on the usage, profile and preferences of individual users [49]. Survey data are a poor proxy, as people with particular demographics and interest profiles will be exposed to different advertising, creating the likelihood of significant error in this analysis.

Finally, we note that, given that 19% of the UK population are under 16 [61], and UK children are active users of the internet [34], the estimated 5.6% HFSS advertising seen by children appears very low. Ofcom estimates children’s weekly digital media use with three metrics (internet average 15 h; gaming 10 h; mobile 10 h). These may sum up to a maximum of 35 h weekly, although these times may overlap, and how much of this time is spent in settings that generate advertising exposure is not clear. In comparison, UK adults’ weekly online average use is 24 h [62]. Note that this includes working hours during which one can assume that most adults work with applications that are not advertising-heavy. Therefore, it appears reasonable to infer that UK children use online applications with advertising more than adults. In this context, applying just 5.6% of advertising to children’s viewing seems quite low.

Overall, therefore, we conclude that industry data review indicates that, throughout the Government Model, the food marketing spend, as well as the likely food and drink splits, likely HFSS splits and likely child HFSS impressions were substantially underestimated.

## 4. General Discussion

This study aimed to assess the validity of the UK government 2019 Impact Assessment’s unprecedented method developed by Kantar Consulting (the “Government Model”) for estimating children’s exposure to digital marketing online. It did so via a targeted review of industry, grey and academic literature with the specific goal of identifying data and resources to test the assumptions and data in the Model. The analysis concludes that: (a) the Government Model consistently and very substantially underestimates online advertising spend, as a result of the methods and data sources chosen; and (b) industry spend data poorly reflect digital marketing activity, further depressing the likely total exposure figure. What are the implications of these findings for understanding children’s actual unhealthy food marketing exposure, for children’s health and well-being, and for public policy?

### 4.1. The Actual Scale of Exposure

As this paper has spelled out, it is particularly challenging to estimate the *actual* scale of digital unhealthy advertising that children are likely to see on digital devices. There are no studies to date measuring children’s marketing exposure in the UK.

#### 4.1.1. Estimating the Scale of Exposure

Given the many uncertainties about data in the digital sphere we have discussed extensively above, we are cautious about speculating what the exact factor of the Government Model’s underestimate might be. However, its conclusion leads the Department of Health to assert that UK children’s annual online ad exposure is one-fifth of their TV ad exposure, 0.73 billion impressions versus 3.6 billion. In contrast, the Dentsu Aegis Network Global Ad Spend report in 2019 records UK digital ad spend as three times the TV ad spend [63], thus a difference of a factor of 15. Without considering the many ways in which this paper explains how digital ad spend underaccounts for reach, applying this proportion would result instead in 10.95 billion online impressions.

Turning to the industry data, we draw on our analysis; these indicate a 3-fold underestimate for Question 1 and an 8-fold underestimate for Questions 2 and 3. (For Questions 4–7, the factors are unknown, but our analysis indicates that all are likely to be underestimated.) Remaining conservative by applying the factors for Questions 1 and 3 alone suggests the possibility of a 24-fold underestimate, or over 17.5 billion impressions. These very rough estimates suggest that the Impact Assessment [19] may underestimate the scale of online marketing of unhealthy food to UK children by a factor of 15–25, if not more.

Are there factors that might depress this? The Impact Assessment (p. 126) notes that it has not factored in the existence of self-regulatory policies for unhealthy marketing to children in the UK since 2017 [19]. What is the likely impact of this self-regulation?

#### 4.1.2. Self-Regulation and Its Limits

The UK’s Advertising Standards Authority (ASA) Advertising Code from the Committee of Advertising Practice was updated in 2017 [64]. It bans ads for HFSS products in “children’s media” online. It permits them where media target a general audience (although prohibiting ads condoning poor nutritional habits, an unhealthy lifestyle in children, promoting pester power, or misleading about nutritional or health benefits; p. 2) [64].

Evidence from other media suggests that the emphasis on channels considered “clearly child-focused” is likely to miss much of children’s actual exposure: as has been demonstrated consistently for television [65,66], a great deal of children’s engagement with online content is in cross-over territory such as gaming, entertainment and sports that appeal to mixed audiences.

The ASA itself has not been able to carry out an assessment of children’s exposure due to the complexity of the digital marketing ecosystem [64]. However, they created automated “avatars” that crawled the web using data profiles that mimicked children. These identified 947 Code breaches in two weeks on “clearly child-focused” YouTube Channels (p. 28). In 2020, the ASA reported continued breaches of the Code [67] with an online sweep of sites. Crucially, these ASA studies focused on sites attracting disproportionately high child audiences and did not include assessments of exposure in social media.

The evidence to date regarding self-regulatory schemes is that their terms are lax, with a focus on paid advertising and on sites predominantly directed at children—yet both on TV and online, children make extensive use of mixed-audience settings [2,10,65,66]. As a result, the impact of regulation on children’s actual exposure is typically limited.

In sum, evidence for children’s continued exposure to digital advertising for unhealthy foods, and the very significant scale of the underestimate of this Impact Assessment, lead us to conclude that digital advertising is the most significant paid-for media channel for unhealthy advertising to children. Furthermore, as we detail above, its reach is likely to extend far beyond what it has paid for.

### 4.2. Implications for Health and Well-Being

The negative effects of food marketing on children’s food preferences, purchases and consumption, and hence on health, are well established and well evidenced [2,3,4,5,6,7,8,9,10].

Although industry evidence for ad effectiveness is generally considered commercially sensitive, some evidence for digital marketing effects is publicly available. In France and the USA, the direct return on investment for digital Coca-Cola and Cadbury campaigns is reported to have been about four times greater than for television campaigns; e.g., in a Coca-Cola campaign in France, Facebook accounted for 2% of marketing cost but 27% of incremental sales. Facebook ads in 14 campaigns generated nearly triple the ad recall as compared with control groups; and econometric analysis of fast-moving consumer goods brand marketing (including food and drinks) in Europe found that combining digital marketing with other media magnified returns on television (by 70%) and on cinema (by 71%) [2].

Adolescents are particularly heavy users of digital media [34]. Assumptions are often made that, as adolescents have advertising literacy, i.e., they understand the commercial, persuasive purpose of advertising, they are therefore able to make food choices to resist its effects [2,10,68,69,70]. Although teens are increasing in agency and autonomy and are certainly well versed in the techniques used by advertisers in these important social spaces, they are not immune to the powerful impact of marketing, and this is particularly the case online. Advertising online, including food marketing, targets adolescents specifically, drawing successfully on their social-developmental needs for connection with peers, working under the radar to activate emotional, identity-laden responses and build long-lasting relationships with brands [35,36,71,72,73]. Buchanan and colleagues [74] note that “earned” social media advertising (forwarded by users within networks—see Box 1) is believed to have the most detrimental effects on young consumers, as this form of marketing is no longer clearly commercial, and can be disguised as a post by the user. Indeed, industry data suggest that “native” advertising (particularly when delivered within social media news feeds) generates positive reactions compared to more overt forms of display advertising, resulting in a 28% increase in advertising viewed on mobile devices and a greater subconscious reaction [75].

Teens aged 13–15 years in Norway described believing that YouTube influencers’ promotion of products was genuine and (ironically) free of any influence [76]. Experimental studies show that after viewing food marketing activity by an influencer popular with young people, compared to when the same influencer promotes non-food, children eat more snacks [77].

Unhealthy food brands can also play a role in adolescents’ social relationships and identity creation: Murphy and colleagues [71] found that teens prefer social media HFSS advertising posts to those for healthy items or non-food items and are more likely to share, or judge a peer positively, if HFSS advertising posts are in their feed.

Finally, we note that in the COVID era, the scale and tactics of digital food and drink marketing globally require urgent further review, at a time when, due to lockdowns, quarantines, and school closures, children are using digital media more than ever before. In 2020, as the pandemic grew, there were initial reports of ad spend retrenchment [78,79] but subsequently of upswings in consumer interest in food and eating and strong brand performance [80]. Brands were advised to “build salience and mental availability” [81], and many food and drink brands did so by capitalising on emotive COVID-related themes of boredom, comfort and being “in this together” [82]. Brands rapidly developed promotions, e-commerce and increased product availability online in nearly 800 recorded campaigns across 90 countries [83]. In the context of risks of poor COVID outcomes presented by obesity and other co-morbidities associated with the excessive consumption of unhealthy foods, these strategies are particularly egregious.

### 4.3. Implications for Public Policy

The clear implication for public policy of marketing for unhealthy products to which children are exposed at scale online is that such marketing should cease. The current UK proposal [19] is for a watershed banning advertising of unhealthy products online before 9 p.m. How are proposed restrictions defined? How effectively will they be implemented? How is impact conceptualised? Although a close consideration is beyond the scope of this paper, we note the following issues with the UK proposal.

#### 4.3.1. Product Versus Brand Advertising

The UK proposal would restrict *product* advertising—but not *brand* advertising. Brand advertising is a cornerstone of building, maintaining and expanding a market and sales. HFSS products themselves need not be featured for them to be effectively advertised; use of the brand is a “shortcut” visual device whose power lies in being able to activate consumer emotions and desires [84,85,86,87].

#### 4.3.2. Lax Nutrient Profiling

A second concern regarding the proposed regulation is that the UK’s nutrient profiling model is lax compared to other international examples in the products it permits to be advertised [75,76]. For example, of a set of television food and beverage advertisements shown at high child-viewing times, UK nutrient profiling permitted half of these, whereas the World Health Organization’s European Region Nutrient Profile Model only permitted a quarter [88,89].

#### 4.3.3. A “Watershed” Concept in Digital Media

Third, the Impact Assessment itself [19] notes difficulties with a “watershed” (time-limited) model for much online marketing, including social media, viral and influencer marketing and advertisers’ own websites (pp. 35–36). Indeed, many of the advertising, marketing and brand activation strategies we highlight in this paper would evade restrictions of “advertising” altogether. These include influencer marketing, event sponsorship, brand marketing and others.

#### 4.3.4. How Impact Is Conceptualized

The Impact Assessment projects calories not consumed by children (based on short-term experimental interventions with children); weight not gained; and consequent health benefits. This is a narrow conceptualisation of impact. It is likely that reduced marketing would benefit not only children but also adults [90]. Other potential benefits include a likely shift in normative conceptualisations of consuming highly processed foods [91], improvements in overall diet quality and a range of social and environmental benefits [92].

#### 4.3.5. Additional Harms Beyond Health

There are further harms that flow from digital marketing practices [12]. The widespread extraction of personal data breaches children’s privacy rights, and the use of these data and of the design affordances of digital media to extract attention and engagement constitutes a form of behavioural manipulation, infringing on children’s right to be free from exploitation, as we argue elsewhere [12,93]. Any complete assessment of the impact of food marketing on children’s well-being also needs to take this manipulation of their selves, identities and social relationships into account.

### 4.4. Current Developments

This field is developing rapidly [10]. In August 2020, the UK government announced that it proposed to implement digital food marketing restrictions in the form of a 9 p.m. watershed by 2022, and that it would consult on a total ban [94]. In the same month, Google announced food marketing restrictions in the UK and the European Union except to users declared over 18 years, to be implemented from October 2020 [95].

The impact of these proposals remains to be seen. The analyses we present here highlight the importance of calculating the base of children’s exposure accurately, and of carrying out similar analyses in other countries in order to develop richer data about the unhealthy food marketing environment in which children live across the world.

## 5. Conclusions

The UK 2019 Impact Assessment claimed considerable potential impact of its proposed advertising restrictions for unhealthy foods in digital media. Yet the analyses presented here show that the likely impact would be much greater, as the Government Model developed by Kantar Consulting underestimated the scale of the market in every one of its seven steps. It therefore very substantially underestimated the scale of UK digital advertising for unhealthy foods—at least tenfold, if not substantially more. This has substantial implications for children’s exposure to unhealthy food marketing, and hence for their health and well-being. We recommend that future Impact Assessments attend to the full scale and scope of marketing and its effects. Only then will it be possible to identify the full range of benefits of restricting marketing to children in digital media.

## Figures and Tables

**Table 1:** Summary of UK Government Model’s assessment of children’s digital advertising exposure to unhealthy food, calculated from ad spend.

Assumptions and Questions	Review and Findings
*Base Assumption:* Digital marketing exposure can be estimated from industry spend data.	Spend metrics poorly reflect the scale and reach of digital advertising. Finding: Digital ad spend is mistakenly equated with reach
*Question 1: Of all-channel food and drink advertising, what proportion is spent on online/digital?* Digital food advertising is 8%; Digital drink advertising is 5%. Source: Nielsen/WARC	Not supported by other sources—underestimated by a factor of 3 or more. Q1 Finding: The scale of digital marketing is underestimated
*Question 2: Of UK advertising spend overall, how much is spent on food and drink?* Food: £927 m (5% of all-channel spend); Drink: £314 m (1.6% of all-channel spend) Government Model sources: Group M and Statista	Likely underestimated—omits retail grocery and restaurants. Q2 Finding: Food and drink ad spend is underestimated
*Question 3: What is the digital food and drink advertising spend total for the UK?* Take the proportions from Question 1 and multiply by the spend data from Question 2 (8% and 5% digital advertising for food and drink). Food: 8% of £927 m = £74 m; Drink: 5% of £314 m = £15.7 m. Government Model result: Estimate UK online food/drink spend as £89.7 m	Question 1 data are faulty estimates of the digital market proportion, not supported by other credible sources. Question 2 data omit aspects of advertising for unhealthy products. Q3 Finding: Digital food and drink ad spend is therefore substantially underestimated
*Question 4: What digital formats does food and drink advertising consist of?* Apply digital advertising format proportions for all sectors to food and drink advertising.	Food and drink advertising spend skews heavily towards display, particularly native, not search Q4 Finding: The digital advertising format most frequently used by food and drink is miscalculated
*Question 5: How many food and drink digital ad “impressions” are there in total?* Divide the overall advertising spend by the price of ads to estimate the number of ads. Do this using the spend splits applied under Question 4; divide by CPM (Cost per Mille) Government Model result: Estimate 22 bn UK digital food/drink ad impressions	- Question 4 assumption is inaccurate. - CPM is fading in use, compared to CPC (Cost per Click) - Food and drink favours native ads (16 x cheaper than banner display) in social media. Q5 Finding: The total number of food and drink ads is underrepresented
*Question 6: How many UK digital HFSS ads are there?* Desktop banner display data show that 59% of food and drink ads are HFSS. Apply this to all digital food and drink ads. Government Model result: Estimate 13 billion digital HFSS impressions	Desktop banner display ads just 9% of digital market; food/drink advertising favours social media, i.e., native and display; ads in mobile (which young people favour) differ from desktop. Q6 Finding: The number of unhealthy ads is underrepresented
*Question 7: What proportion of UK HFSS digital ads are seen by children?* Apply panel survey data to estimate proportion of HFSS impressions seen by children: 5.3% Government Model source: Kantar CrossMedia tool. Final Government Model result: Estimate 0.73 bn HFSS impressions seen by children in UK annually	CrossMedia Kantar panel survey data assume generic viewing patterns—yet digital advertising is targeted. Also, 19% of the UK population are under 16 years and are active internet users; estimating 5% views appears very low. Final finding: Child exposure to unhealthy ads is very substantially underrepresented, by at least a factor of 10.
